# Bi-sigmoid spike-timing dependent plasticity learning rule for magnetic tunnel junction-based SNN

**DOI:** 10.3389/fnins.2024.1387339

**Published:** 2024-05-15

**Authors:** Salah Daddinounou, Elena-Ioana Vatajelu

**Affiliations:** TIMA, Grenoble INP, Univ. Grenoble Alpes, Grenoble, France

**Keywords:** SNN, STDP, neuromorphic, MTJ, spintronics, unsupervised, online learning

## Abstract

In this study, we explore spintronic synapses composed of several Magnetic Tunnel Junctions (MTJs), leveraging their attractive characteristics such as endurance, nonvolatility, stochasticity, and energy efficiency for hardware implementation of unsupervised neuromorphic systems. Spiking Neural Networks (SNNs) running on dedicated hardware are suitable for edge computing and IoT devices where continuous online learning and energy efficiency are important characteristics. We focus in this work on synaptic plasticity by conducting comprehensive electrical simulations to optimize the MTJ-based synapse design and find the accurate neuronal pulses that are responsible for the Spike Timing Dependent Plasticity (STDP) behavior. Most proposals in the literature are based on hardware-independent algorithms that require the network to store the spiking history to be able to update the weights accordingly. In this work, we developed a new learning rule, the Bi-Sigmoid STDP (B2STDP), which originates from the physical properties of MTJs. This rule enables immediate synaptic plasticity based on neuronal activity, leveraging in-memory computing. Finally, the integration of this learning approach within an SNN framework leads to a 91.71% accuracy in unsupervised image classification, demonstrating the potential of MTJ-based synapses for effective online learning in hardware-implemented SNNs.

## 1 Introduction

The current landscape of computing, dominated by traditional Von Neumann (VN) architectures, faces significant challenges when it deals with Artificial Intelligence (AI) applications (Ye et al., 2021; Momose et al., 2020). VN architectures which are based on the separation between processing and memory, suffer from substantial energy consumption and computational latency due to the data transfer overhead between the memory and the processor unit (Ma et al., 2020; Petrenko and Petrenko, 2018). On top of that, VN architectures are not the best candidates for IoT and edge-computing intelligent devices because they don't allow online and unsupervised learning Syed et al. (2024). These two characteristics though are important for systems that are intended to learn continuously and adapt themselves in real-time, like autonomous vehicles. In contrast to this architecture, Neuromorphic Engineering, a concept introduced by Carver Mead (Mead, 2020) in the early nineties, has emerged as a promising alternative. This approach, inspired by the biological brain's structure and function, offers a distributed processing model. The brain's computational model operates through a vast network of neurons interconnected by synapses, each capable of processing and storing information. This decentralized approach allows for efficient parallel processing. Neurons communicate via electrical impulses or 'spikes'. The strength of connections, or the synaptic weight, changes in response to the patterns and timings of neuronal activity. This dynamic adaptability, known as synaptic plasticity, is fundamental to learning and memory in the brain. Unlike VN architectures, the huge number of neurons and synapses where processing and memory are colocalized leads to highly efficient computation with minimal energy consumption. Translating the brain's computational principles into artificial systems has led to the development of various systems that all aspire to reproduce the synaptic plasticity of the brain. These systems range from nanowires-based networks (Caravelli et al., 2023; Loeffler et al., 2023; Milano et al., 2022) to Spiking Neural Networks (SNNs), which are considered the third generation of Artificial Neural Network (ANN) models (Maass, 1997; Ghosh-Dastidar and Adeli, 2009). The SNN encodes information in the timing of spikes, and utilizes a dedicated learning rule: Spike-Timing-Dependent Plasticity (STDP) (Caporale and Dan, 2008), that modulates synaptic strengths, either strengthening or weakening, based on the relative timing between spikes. While the SNN model with STDP promises a more energy-efficient solution for AI applications and enables online and unsupervised learning, its practical implementation extends beyond the model and the algorithm itself. The effectiveness and energy efficiency of SNNs largely depend on the appropriate hardware implementation. It is this hardware, especially when designed with in-memory computing, that unlocks the full potential of SNNs, ensuring energy efficiency and brain-like computation.

The state-of-the-art SNN hardware implementations can be split into three categories: first, systems like CPUs, GPUs, and TPUs focused on computational complexity with high accuracy but high power use (Baji, 2017; Wang et al., 2020, 2019); second, power-efficient CMOS-based engines such as TrueNorth (Merolla et al., 2014) and Loihi (Davies et al., 2018), which are constrained by memory bottlenecks; and finally, biologically plausible in-memory computing using non-volatile technology, yet lacking online learning capabilities (Maranhão and Guimarães, 2021; Lone et al., 2022). These approaches struggle to balance energy efficiency with the capability for online unsupervised learning.

Our work aims to address this challenge with an innovative design of synapses using Magnetic Tunnel Junctions (MTJs) Ikeda et al. (2010). These non-volatile and energy-efficient devices have the potential for unsupervised learning through dynamic conductance adjustments, guided by an innovative learning rule. Although there are a few works that proposed the use of MTJs for plasticity dynamics in SNN (Shreya et al., 2020; Jang et al., 2021; Leonard et al., 2022), there is a need to demonstrate this in a full network trained with a device-specific learning rule. *The key contribution of this paper focuses on the synaptic design and a compatible learning rule*. We particularly explore the use of a compound synapse made of multiple parallel-connected MTJ devices, these two-state devices lead to a multi-state synapse thanks to their inherent stochasticity. We carry an extensive design space exploration of the MTJ-based synapse to find the optimal parameters that allow the unsupervised adjustment of the synaptic conductance through a learning rule that is rooted in the physics of the MTJ, we labeled it Bi-sigmoid Spike Timing Dependent Plasticity (B2STDP). This rule gave a good accuracy (>90%) in an image classification task.

The remainder of this paper is structured as follows: Section 2 reviews current SNN hardware implementations and their training approaches. Section 3 details the MTJ-based synapse design, and various design choices that enable the Bi-sigmoid STDP learning rule. Section 4 presents our results and discussions on the implemented network and its performance. The paper concludes with a recap and perspectives in Section 5.

## 2 Related works

The state of the art in SNN hardware implementations is extensive, with various approaches and investigations underway. Pfeiffer et al. (2018) note a significant disparity between the potential efficiency of SNNs and their actual implementations on existing computing hardware. This is attributed to the contrast between the highly parallel and sparse communication nature of SNNs on one hand, which rely on in-memory computation, and the sequential and centralized processing capabilities of CPUs and GPUs on the other hand (Pfeiffer and Pfeil, 2018). To address this challenge, massively parallel digital architectures for SNNs have been proposed. IBM's TrueNorth, consisting of 1 million neurons connected by 256 million synapses, is primarily used for inference after offline training (Merolla et al., 2014). Similarly, the SpiNNaker chip demonstrates efficient performance with 18 cores, approximately 1K neurons, and 1K synapses per core (Furber et al., 2012). The emergence of non-volatile memories, such as memristors (Mazumder et al., 2012), has enabled disruptive implementations of SNNs (Zhao et al., 2020). Querlioz et al. (2013) demonstrated a network-level architecture where synapses utilize memristive devices based on conductive filaments, while CMOS-based neurons with inhibition and homeostasis are employed. The authors affirm that their architecture exhibits resilience against parameter variability (Querlioz et al., 2013). Zhang et al. (2016) propose an All-Spin Artificial Neural Network (ASANN) that employs spintronic devices for both synapses and neurons. They introduced the Compound Spintronic Synapses (CSS) composed of stacked MTJ devices and the Compound Spintronic Neurons (CSN) with multi-step transfer functions. The network is trained offline, followed by weight-mapping to the discrete resistance states of CSSs (Zhang et al., 2016). Regarding the training approaches of SNNs, they can be categorized into three main approaches. Firstly, a strategy that consists of training a traditional neural network and then converting its parameters to operate with spiking neurons, a technique validated by the work of Rueckauer et al. (2017). This approach leverages the capability of simpler spiking neuron models to emulate functions such as the ReLU activation, found in traditional networks, as illustrated by Cao et al. (2015). Secondly, backpropagation (BP) is directly applied to SNNs for training. This method faces the challenge of the non-differentiable nature of spiking neurons' activation functions, typically modeled by a Heaviside function. Researchers have proposed using surrogate gradients or differentiable approximations to address this, as discussed in studies by Lee et al. (2016), Neftci et al. (2019), and Mostafa (2017). Although BP is a second-generation technique that departs from the bio-inspired origins of SNNs, it offers a path to efficient training. The third approach consists of STDP which adopts a more biologically inspired learning mechanism, potentially providing a more natural method for training SNNs. Diehl and Cook (2015) presented a network for digit recognition that has the following features: conductance-based synapses, STDP with time-dependent weight change, lateral inhibition, and adaptive spiking thresholds. The architecture of their network which consists of an input layer, excitatory and inhibitory neurons layers has inspired a lot of other works. Their unsupervised learning scheme achieves 95% accuracy on the MNIST dataset (Diehl and Cook, 2015). While many hardware implementations of SNNs focus on inference, online training remains a big challenge because of the lack of implementations that can dynamically update their weights in response to the changing environments that they are learning. Maranhão and Guimarães (2021) proposed a toy model with post-synaptic neuron spiking and memristive synapses that update in one direction only. Similarly, Andreeva et al. (2020) used different CMOS neurons for input and output with memristive synapses. However, both implementations lack proper time-dependent STDP. Our work addresses this gap by introducing a hardware implementation of STDP with clear time dependence which is suitable for SNNs. We demonstrate the careful selection of signal shapes to emulate pre- and post-synaptic spikes, enabling the implementation of STDP in hardware (Daddinounou and Vatajelu, 2022). In their study, Li et al. (2014) focused on the device level and designed voltage pulse schemes, using different voltage shapes to achieve STDP in chalcogenide memristors, by leveraging the gradual resistance of the synaptic memristor in the microseconds range. They experimentally demonstrated four different STDP curves, representing symmetric/asymmetric Hebbian/anti-Hebbian learning rules. No full network performance was evaluated, and the efficiency of the proposed learning rules is still to be studied (Li et al., 2014). To simulate their SNN, Kim et al. (2021) integrated the behavior of memristors into an existing SNN simulator which uses rate coding and the architecture from Diehl and Cook (2015). They concluded that SNNs exhibited strong tolerance for weight-update non-linearity, with network accuracy remaining relatively high under two conditions: symmetric Long-Term Potentiation (LTP) and Long-Term Depression (LTD) curves, or positive non-linearity factors for both LTP and LTD (Kim et al., 2021). Finally, Garg et al. (2022) focused on the training algorithm and suggested an alternative to STDP; the Voltage-Dependent-Synaptic Plasticity (VDSP) rule which uses the decaying membrane potential of the presynaptic neuron to estimate when it has fired and updates the synaptic weight accordingly when the postsynaptic neuron fires, this method and the one we propose here reduce the number of updates by half compared to classic STDP since the update happens only at the postsynaptic firing Garg et al. (2022).

## 3 Materials and methods

In this section, we aim to demonstrate the suitability of MTJ-based synapses for enabling unsupervised on-chip learning in SNNs. To achieve this, we first provide an overview of the fundamental working principle of MTJs, then we highlight their significance as building blocks for synapses. Furthermore, we introduce an adapted STDP rule tailored specifically for this MTJ-based design. Subsequently, through extensive SPICE simulations we delve into the justification and discussion of various design choices that were made.

### 3.1 STT-MTJ device

**Figure 1:**
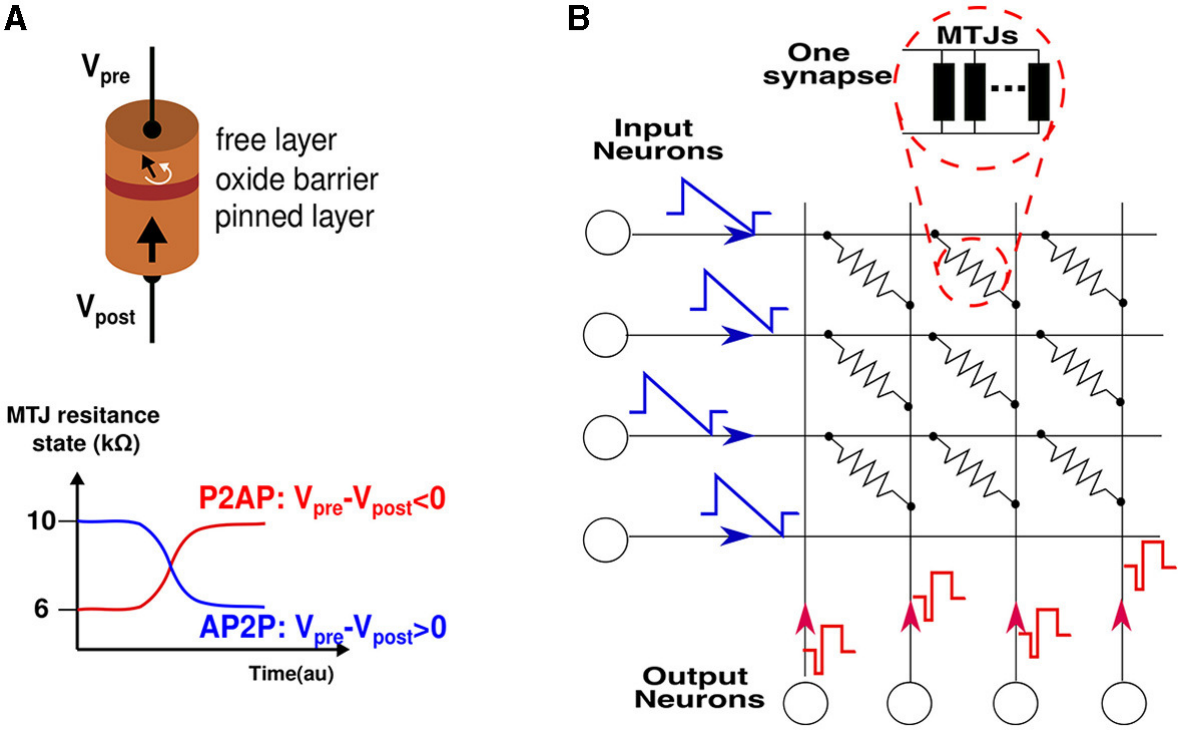
**(A)** A schematic of an MTJ device composed of two ferromagnetic layers which are separated by an insulating layer. on the right is shown the switching mechanism from parallel to anti-parallel (P2AP) and vice versa (AP2P) depending on the current polarity. **(B)** An SNN network composed of the synaptic crossbar, input, and output neurons emitting their specific pulses. Each synapse is a set of multiple MTJs connected in parallel to enable a multi-level conductance synapse.

STT-MTJ (Spin Transfer Torque Magnetic Tunnel Junction) is a nanoscale electronic device that utilizes the phenomenon of spin transfer torque to control the magnetization state of a magnetic tunnel junction (Xu et al., 2008). It consists of a structure composed of two ferromagnetic layers separated by a thin insulating barrier. Of these layers, one is fixed, while the other has the freedom to rotate its magnetization direction. This rotation can align the free layer's magnetization either parallel or antiparallel to the fixed layer's magnetization. This alignment results in two distinct resistive states: a low-resistance state when the magnetizations are parallel (P) and a high-resistance state when they are antiparallel (AP). A schematic of an MTJ is depicted in Figure 1 with the two switching mechanisms of the free layer: from anti-parallel to parallel (AP2P) and vice versa (P2AP). The operation of STT-MTJ relies on the injection of a spin-polarized current from the fixed layer into the free layer. Due to the transfer of angular momentum from the current to the magnetization, a torque is exerted on the free layer, influencing its magnetization orientation. This torque can either assist or resist the magnetization-switching process depending on the direction and polarization of the current. By manipulating the magnitude, direction and duration of the injected current, it is possible to control the magnetization state of the free layer in the STT-MTJ. This ability to toggle the magnetization between two stable states (parallel and antiparallel) forms the basis for its application in non-volatile memory. STT-MTJs offer several advantages including low power consumption, high switching speeds, high endurance, and scalability to small device dimensions. These properties make STT-MTJs attractive for various spintronic applications, such as Magnetic Random Access Memory (MRAM), magnetic sensors, and neuromorphic computing systems. The switching process in the MTJ is governed by two regimes. The first is known as the precessional regime (MTJ described by the physics of Sun model), characterized by fast and deterministic switching that necessitates a high current. The second regime, known as the probabilistic regime (MTJ described by the physics of the Neel-Brown model), exhibits slower and probabilistic switching, requiring relatively lower currents. In this regime, the reversal of magnetization in the free layer, thus switching the state, is thermally assisted. This behavior is captured in an accurate compact model described in VerilogA (Zhang et al., 2015) to run electrical simulations, we use this model to compose the synapses and characterize the spintronic compound.

### 3.2 Compound MTJ synapse

**Figure 2:**
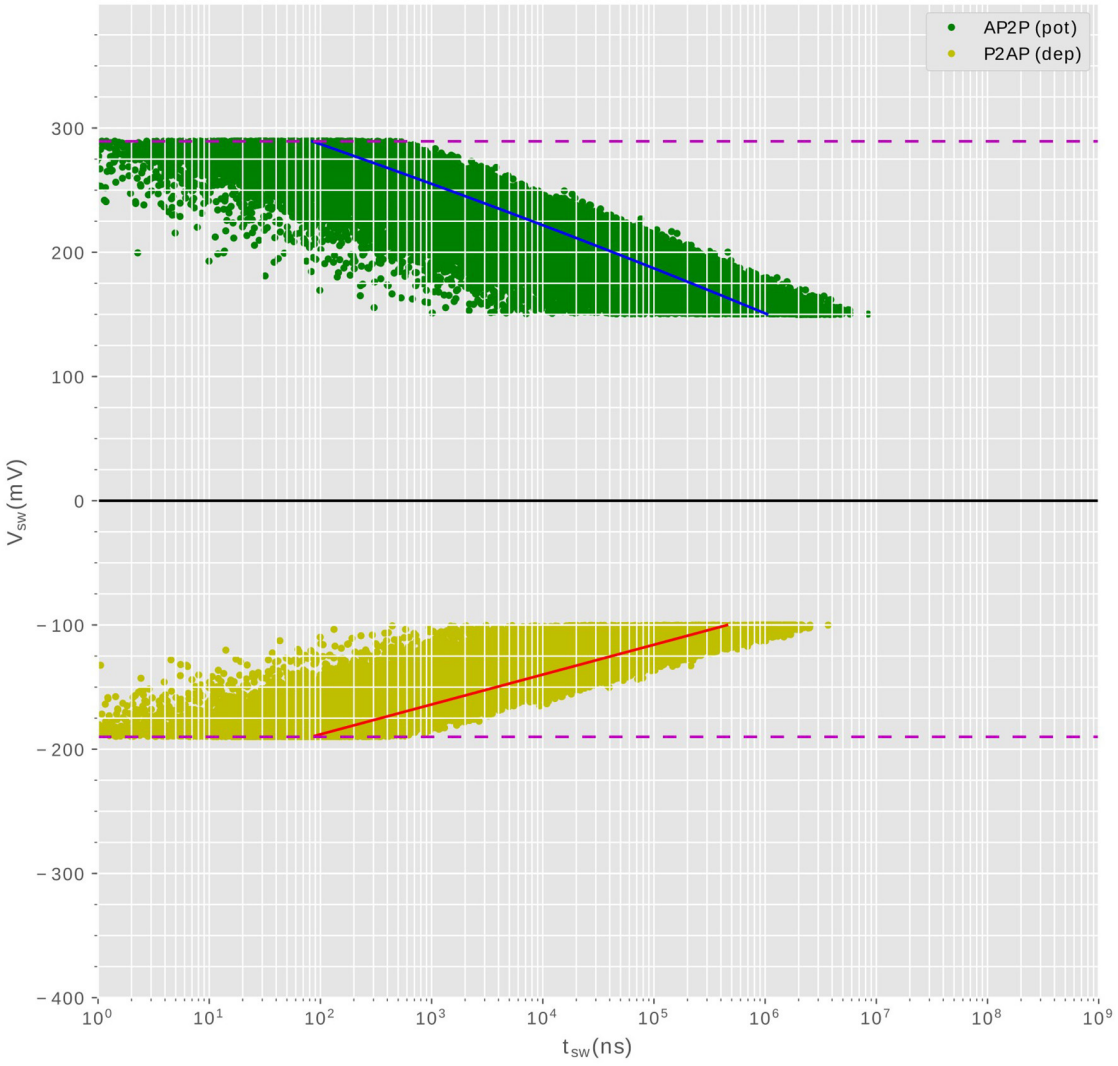
Statistical simulations showing the required voltage (amplitude, duration) to switch the MTJ state for both potentiation (AP2P) and depression(P2AP). For each voltage amplitude, we run 100 simulations to get the distribution of the required pulse width durations.

The compound synapse is composed of multiple MTJ devices in parallel, with the MTJs used in the probabilistic regime. This choice allows the synapse to exhibit an equivalent conductance within a range of discreet levels, as the intrinsic stochasticity of the MTJ devices ensures that they do not switch all simultaneously when the writing current flows through. Figure 2 shows the result of electrical simulations of an MTJ under the probabilistic regime used to obtain the relationship between the required voltage to trigger state reversal and the pulse width. A large number of simulations are carried out to be statistically relevant. Depending on the polarity of the current, we distinguish between two cases: potentiation (AP2P switching) which increases the conductance, and depression (P2AP) which decreases the conductance. The potentiation is presented in Figure 2 with green points(positive voltage is applied), whereas depression is plotted in yellow points (negative voltage is applied).

Additionally, aside from the current polarity, an asymmetry is apparent in the plot. This asymmetry arises from the relatively less stable equilibrium state of the parallel configuration of the two magnetic layers. As a result, the stability is readily disrupted in favor of the anti-parallel configuration. The (pulse width (W), voltage amplitude (V)) pairs required for state reversal are not unique due to the thermal fluctuations that confer a stochastic nature to the device. Hence, we characterize the MTJ device by conducting multiple electrical simulations to obtain the distribution of (W, V) values required for switching. For both current polarities, a valley of points is obtained, indicating that for a fixed applied voltage, the time required for switching is not constant. The stochasticity manifests itself in non-deterministic pulse widths within a certain range, which implies that the probability of switching increases with increasing pulse width. The synapse is then implemented using multiple MTJ devices in parallel, each MTJ presenting a binary state, but once put together, the resulting compound synapse becomes a multi-level conductance device, which is suitable to tune and then hold the synaptic weight. During training, the STDP learning rule programs the synapse to a given conductance level among the possible levels supported by the design. Figure 1 shows an SNN network to be implemented in hardware. The synapses form a crossbar array, each of them is a compound of multiple MTJs connected in parallel. The input neurons encode the information into spikes, and the output neurons are Leak Integrate and Fire (LIF) neurons (Hunsberger and Eliasmith, 2015), their design is out of the scope of this article.

### 3.3 Learning rule

**Figure 3:**
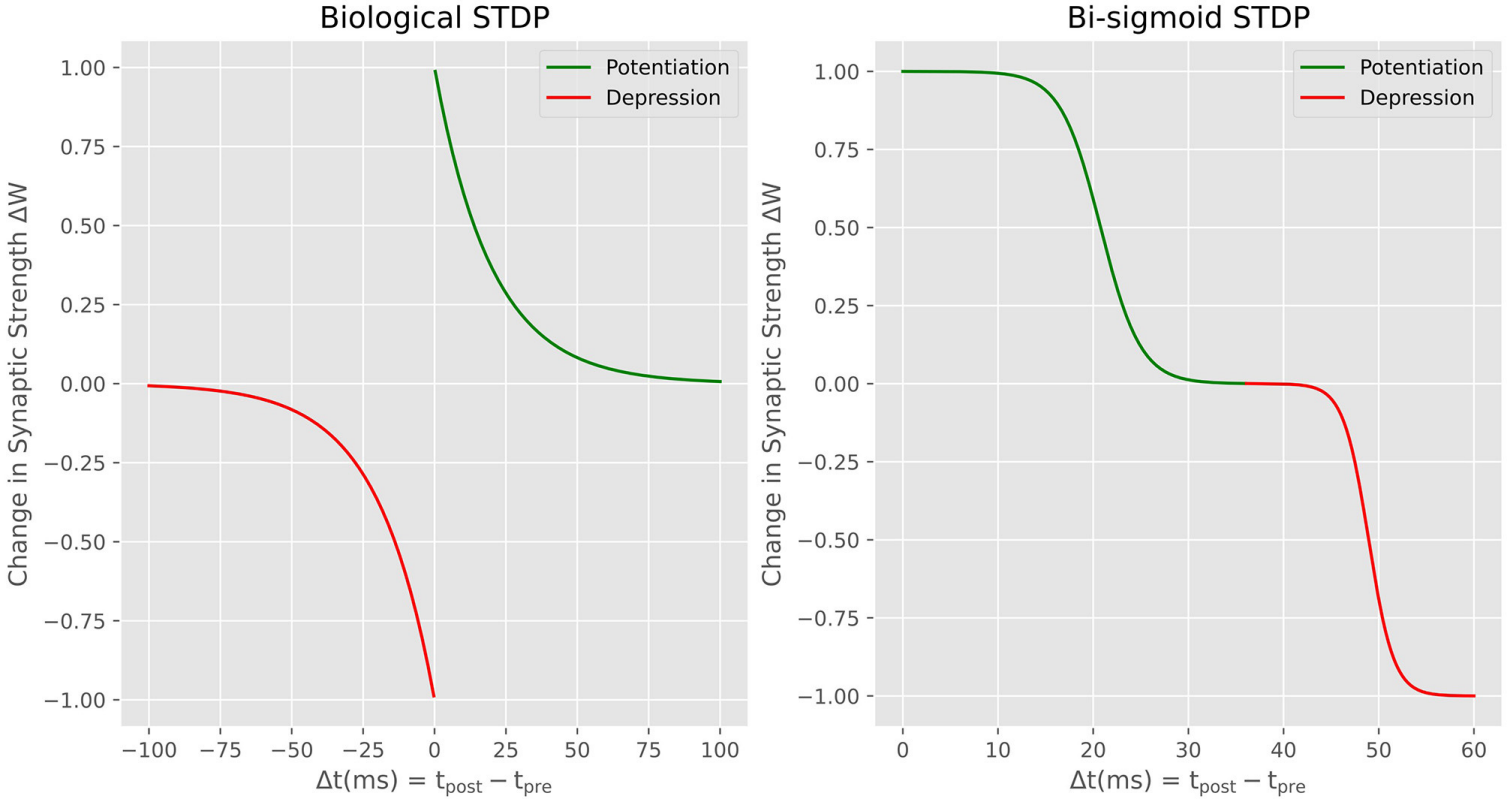
Biological STDP vs. Bi-sigmoid STDP, the weight update in the function of the time difference between pre- and post-synaptic pulses.

The STDP rule is a fundamental mechanism governing synaptic plasticity in the brain. The rule takes into account the relative timing between pre-synaptic and post-synaptic spikes to determine whether the synaptic connection should be potentiated or depressed (increase or decrease the connection strength). Specifically, when a pre-synaptic spike occurs shortly before a post-synaptic spike, the synapse is potentiated. On the other hand, when the post-synaptic spike precedes the pre-synaptic spike, the synapse is depressed. The biological STDP exhibits a decaying exponential relationship between the relative timing of spikes and the weight update for both potentiation and depression as it is shown in the left side of Figure 3. To reproduce this learning rule in neuromorphic hardware, most of the published works introduce an additional variable for each neuron called *a trace* (Diehl and Cook, 2015). The traces store the spiking history of the neuron so that the precise timing of spiking events and the temporal order of pre- and post-synaptic activities is used to compute the corresponding synaptic update. Introducing the trace variable means adding a memory overhead where this variable should constantly be updated for each spike. However, proper local learning doesn't need to fetch either weight values or environment variables such as spiking history elsewhere. The advantage of our proposal is the fact that the STDP is performed locally with no need to access external memory. This is also what have been explored by other works (Prezioso et al., 2016)Covi et al. (2016) interested on reproducing biological STDP in memristive devices. Here We propose *the Bi-Sigmoid STDP* which is a modified version of the classic STDP curve seen in biology. Through this new learning rule that is directly derived from the physics of the MTJ-based synapse, our focus is on preserving the essential functional characteristics of STDP, rather than on an exact imitation of the biological counterpart. The Bi-sigmoid STDP is shown on the right side of Figure 3. It shows no negative temporal part because the update is done on the fly with no access to the spiking history. It simply says that, for a given pre-synaptic spike, if the post-synaptic neuron spikes shortly after, the synaptic weight is increased (potentiation), whereas if the post-synaptic neuron spikes long after the pre-synaptic spike, the weight is decreased (depression).

### 3.4 Design choices

#### 3.4.1 Voltage shapes

**Figure 4:**
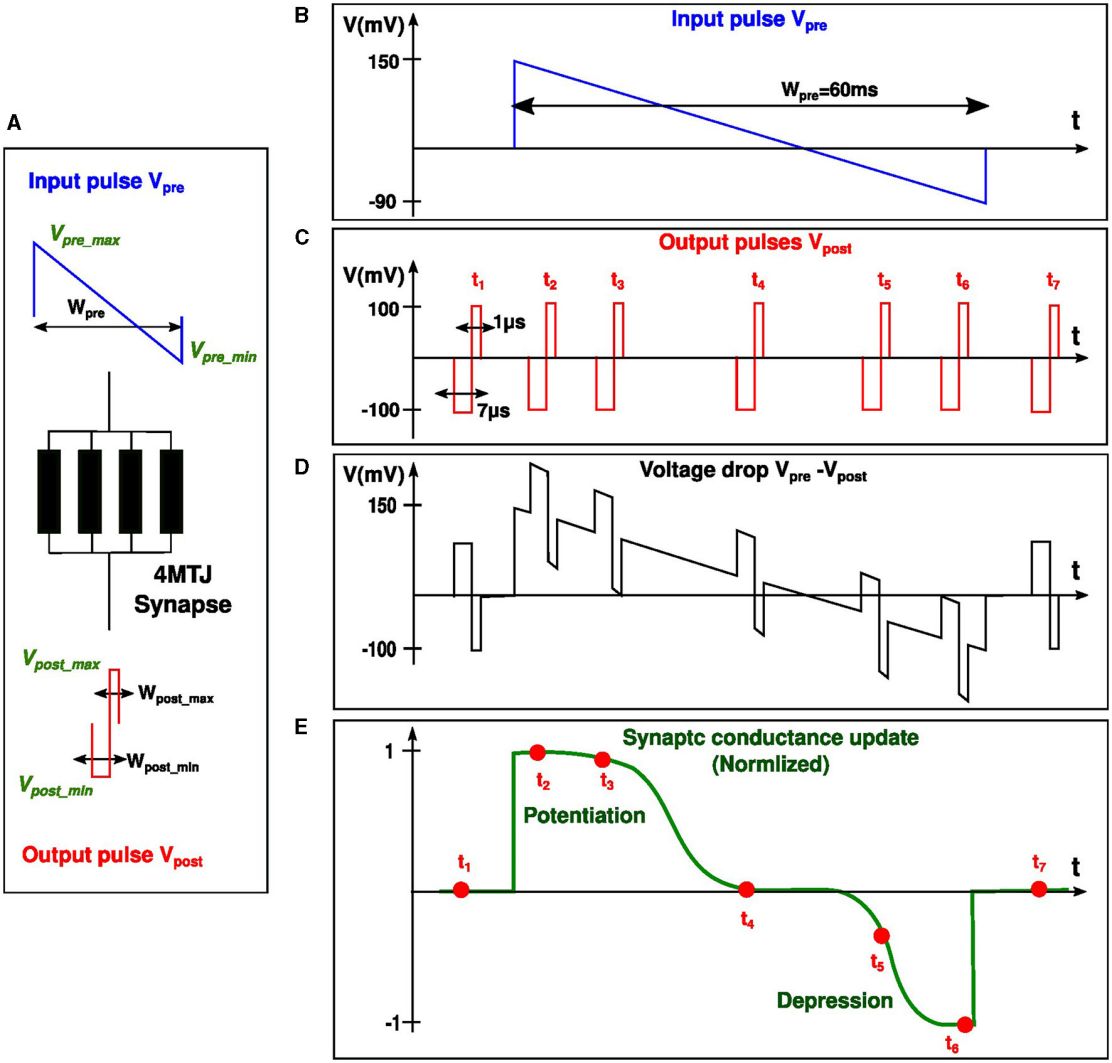
Temporal relationship between pre- and post-synaptic pulses (*V*_*pre*_ and *V*_*post*_), followed by the corresponding synaptic update. **(A)** 4MTJ synapse subject to the voltage drop *V*_*pre*_ − *V*_*post*_. **(B)** and **(C)** depict *V*_*pre*_ and a sequence of *V*_*post*_ respectively. **(D)** shows different scenarios of the resulting voltage drop across the synapse when *V*_*post*_ arrives at different delays (t1 to t7) relative to *V*_*pre*_. **(E)** provides a normalized view of the synaptic conductance update, highlighting periods of potentiation and depression corresponding to the timing sequences t1 to t7.

In order to enable the implementation of the bi-sigmoid learning rule, we developed accurate signal profiles for both pre- and post-synaptic spikes. The signals *V*_*pre*_ and *V*_*post*_ were designed in such a way that the voltage drop across the synapse *V*_*pre*_−*V*_*post*_ over time, starting from the rise of *V*_*pre*_, makes the synapse updating its state according to the bi-sigmoid rule, ie: potentiation at the beginning and depression at the end of *V*_*pre*_ signal. Aligning with the brain's methodology of handling cognitive tasks at frequencies in the kHz range, our design similarly prioritizes energy efficiency over high-speed processing. For this reason, The pre-synaptic signal *V*_*pre*_ lasts for a duration spanning several tens of milliseconds, during which the synaptic update should take place. On the other hand, the post-synaptic signal *V*_*post*_ which lasts only for some microseconds, is the one that triggers the update on the synapse. The amount and direction of the update depends on when *V*_*post*_ arrives relative to *V*_*pre*_. To summarize, the synaptic update happens only when the *short* post-synaptic signal overlaps with a part of the *long* pre-synaptic signal (*see* *Figure 4*).

Since the MTJs of the synapse are operating in the probabilistic regime, we want to assign a high probability for switching to the high conductance state when *V*_*post*_ arrives at the beginning of *V*_*pre*_ (potentiation). Likewise, we want to assign a high probability for switching to the low conductance state when *V*_*post*_ arrives at the end of *V*_*pre*_ (depression). The likelihood of MTJ switching is directly dependent on the amplitude and pulse width of the applied voltage. While the pulse widths are all fixed, and both positive and negative amplitudes of *V*_*post*_ are fixed as well, the only variable left is *V*_*pre*_ amplitude which should take a decreasing voltage shape with a positive part at the beginning which favors potentiation and a negative part at the end which favors depression. Figure 4 demonstrates how the voltage drop *V*_*pre*_−*V*_*post*_ should depend on the delay between the two signals in order to obtain a bi-sigmoid weight update on the synapse, this behavior will be explained in simple steps. As the delay between pre- and post-synaptic spiking increases, we observe five distinct behaviors in the following order:

- *High potentiation:* When the postsynaptic neuron spikes immediately after the presynaptic neuron, the voltage drop *V*_*pre*_−*V*_*post*_>0 reaches its maximum value, causing the conductance of the synapse to increase significantly.
- *Low potentiation:* As the delay increases, the voltage drop remains positive but with a smaller amplitude, resulting in a small increase in the conductance of the synapse.
- *Unchanged conductance:* This is the transitional region between potentiation and depression. The voltage drop decreases, making it unable to produce potentiation. As the delay continues to increase, the voltage drop becomes negative, but it does not cause depression since it has not yet crossed the negative threshold voltage.
- *Low depression:* When a significant delay occurs, the voltage drop *V*_*pre*_−*V*_*post*_ crosses the threshold in the negative direction, causing a drop in the conductance of the synapse.
- *High depression:* If the postsynaptic pulse arrives extremely too late compared to the rise of the presynaptic pulse, the negative voltage drop becomes too big in absolute value, causing a substantial decrease in the conductance of the synaptic connection.

The pre-synaptic input *V*_*pre*_ (Figure 4B) has a duration *W*_*pre*_, a maximum positive amplitude *V*_*pre*_*max*_ and a maximum negative amplitude *V*_pre_min_. Similarly, the post-synaptic pulse *V*_*post*_ (Figure 4C) has two rectangular parts with amplitudes *V*_*post*_*min*_ and *V*_*post*_*max*_, and pulse widths *W*_*post*_*min*_ and *W*_*post*_*max*_. When only *V*_*pre*_ or *V*_*post*_ spikes occur, the voltage drop across the synapse (Figure 4D) is not large enough so it should not modify the synaptic weight *(t*_1_, *t*_7_ *on* *Figure 4E**)*. However, when both spikes occur, the voltage drop across the synapse is given by *V*_*pre*_−*V*_*post*_ (Figure 4D). The negative amplitude of *V*_*post*_ adds up to the positive amplitude of *V*_*pre*_, and if the positive *Brown threshold* is exceeded *(150mV)*, the synapse will potentiate *(t*_2_, *t*_3_ *on* *Figure 4E**)*. On the other hand, If the post-synaptic pulse arrives toward the end of the pre-synaptic pulse, the positive part of *V*_*post*_ is subtracted from the negative part of *V*_*pre*_. If the negative *Brown threshold* is exceeded *(-100mV)*, the synapse will depress *(t*_5_, *t*_6_ *on* *Figure 4E**)*. This way, the potentiation and depression in the synapse is solely dependent on the time delay between input and output neuron spikes. This qualitative explanation of the main ingredients of our proposed STDP has been accompanied by a thorough quantitative study of the effect of each design parameter on the bi-sigmoid STDP curve. This study has been then followed by an optimization process to ensure a design with the most adequate parameters.

Figure 2 is extremely important for design choices, various information can be extracted to help us choose the profiles of signals (pre- and post-synaptic pulses) to be applied across the synapse so that it produces the desired behavior of STDP. It is important to note that the following characterization which has been done for one single MTJ device applies also for the whole synapse which is a compound of N number of MTJ devices in parallel. The reason is simple: as soon as the same voltage is applied, the current circulating in each MTJ device should be the same according to Ohm's law no matter the number of devices in the synapse. The current driven by the neuron should scale with the number of MTJ devices in the synapse though. This affirmation has been verified by electrical simulations. Figure 2 allowed us to extract some design conditions that will be discussed next.

#### 3.4.2 Minimum threshold voltages

Designing the voltage profiles for input and output neuronal spikes starts by investigating the behavior of these voltages on the MTJ. The characterization of the MTJ shown in Figure 2 highlights a specific voltage range that can trigger state switching in the MTJ under the probabilistic regime. This range is defined by a lower threshold voltage and an upper critical voltage, depends of course on the MTJ's geometrical properties. There exist two minimum thresholds, 150*mV* and −100*mV* for potentiation and depression respectively. The *Brown threshold* is the minimal voltage under which no switching should occur, independently from the pulse width. This condition implies that any signal that is not supposed to affect the synapse conductance, during inference for example, should satisfy *V*_*signal*_ < 150*mV* for potentiation and |*V*_*signal*_| < 100*mV* for depression. The pre-synaptic and post-synaptic pulses are not supposed to affect the synapse's conductance neither if they are not synchronized (if there is no temporal overlapping). Hence, *the first design requirement can be given by:*

- *V*_*pre*_*max*_ and *V*_*post*_*max*_ both should be smaller than 150*mV*: to prevent undesired potentiation when *V*_*pre*_ or *V*_*post*_ arrives separately.
- *V*_*pre*_*min*_ and *V*_*post*_*min*_ both should be smaller than |100*mV*|: to prevent undesired depression when *V*_*pre*_ or *V*_*post*_ arrives separately.

#### 3.4.3 Maximum critical voltages

The probabilistic regime of the MTJ device is only defined within an interval, where the MTJ behavior is described by the Neel-Brown model. The upper limit of that interval is different for potentiation and depression due the equilibrium stability issue discussed earlier. Valuable information extracted from Figure 2 suggests that no individual signal nor a combination of the input-output signal should exceed −190*mV* for depression nor 289*mV* for potentiation. This shouldn't happen in any stage of synapse manipulation. If the upper limits were exceeded, the synapse enters the deterministic regime described by the Sun model, where all the MTJs of a single synapse will switch immediately and behave equally, rendering the synapse a two states component, by losing the intermediate states corresponding to the number of MTJ devices. *The second design requirement can be summarized as:*

- 0 < *V*_*pre*_*max*_−*V*_*post*_*min*_ < 289*mV*: to prevent deterministic potentiation.
- 0 > *V*_*pre*_*min*_−*V*_*post*_*max*_ > −190*mV*: to prevent deterministic depression.

We used the following values in this work: *V*_*post*_*max*_ = 100*mV*, *V*_*post*_*min*_ = −100*mV*, *V*_*pre*_*max*_ = 150*mV* and *V*_*pre*_*min*_ = −90*mV*.

#### 3.4.4 Pulse width constraints

**Figure 5:**
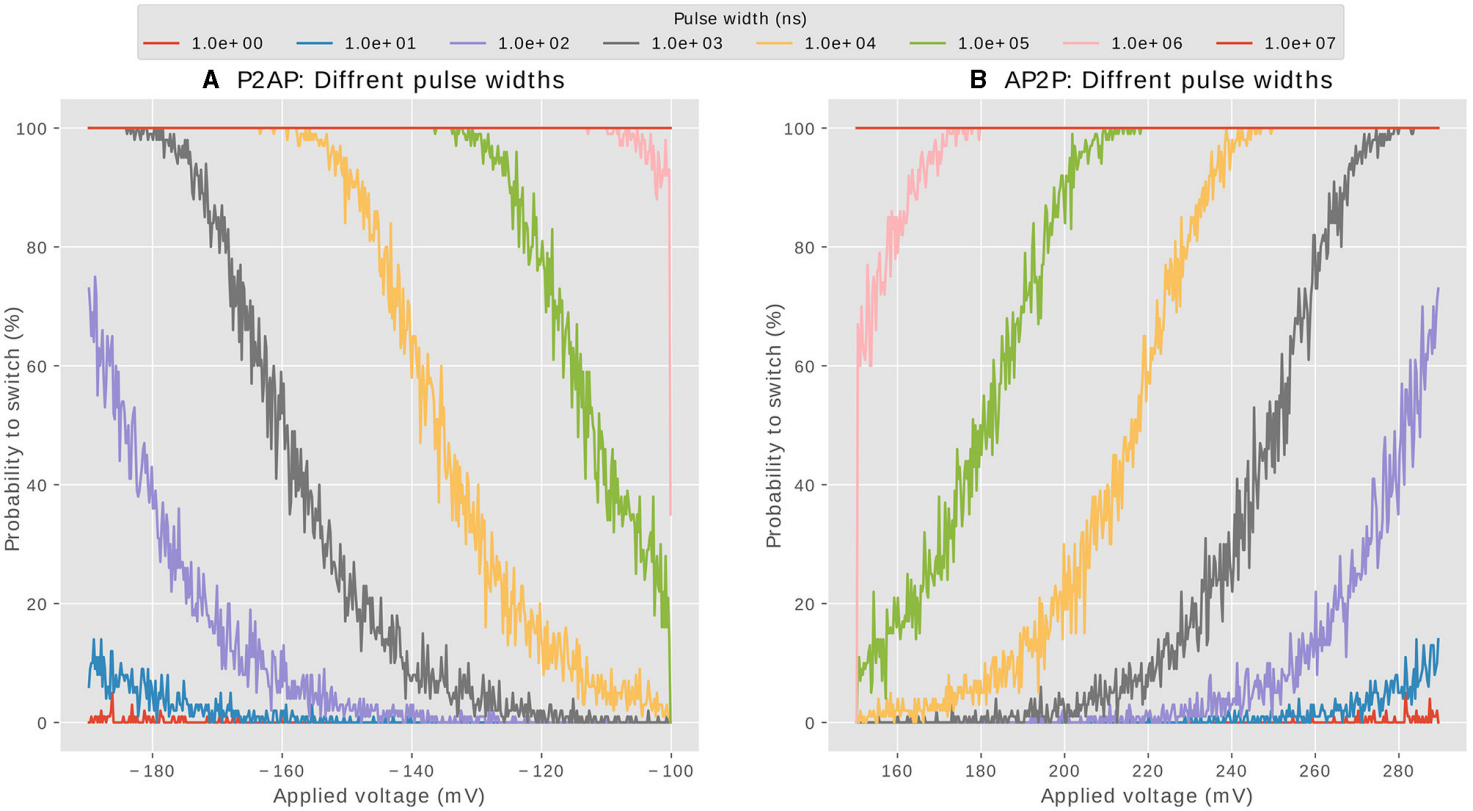
Probability of switching of an MTJ at various pulse widths, when the voltage is varied within the Neel-Brown interval. **(A)**: Depression, **(B)**: Potentiation.

We start by analyzing the pulse widths of the positive and negative parts of the post-synaptic signal *V*_*post*_. The pulse width of an applied voltage (within Neel-Brown limits) determines the probability of state reversal of an MTJ. As an example, take the valley points of potentiation (green points in Figure 2); if a pulse width of 10^7^*ns* were to be applied, all the switching points will be inside that interval, the switching AP2P will happen at 100% for whatever voltage, rending the behavior of the synapse deterministic. Such width should be excluded then. If, on the other hand, the width is very small, say 10*ns* the majority of points are outside that interval, the probability of switching is very small, it even tends to zero close to the Neel-Brown threshold at 150*mV*. A good choice for pulse width should allow the probability of switching *to take all possible values from 0 to 100%* when varying the amplitude of the applied voltage, ie: when *V*_*post*_ arrives at different delays compared to *V*_*pre*_. To investigate what are the possible pulse widths that could be used for the two parts of *V*_*post*_, we show in Figures 5A, B the probability of switching of an MTJ from parallel to anti-parallel(depression) and from anti-parallel to parallel (potentiation) respectively. The probabilities are shown with respect to the applied voltage, for different pulse width values. A proper pulse width of a signal intended to induce synaptic potentiation for example, should allow all these three scenarios based on voltage conditions: At high voltage, achieved when pre-synaptic voltage (*V*_*pre*_) and post-synaptic voltage (*V*_*post*_) arrive simultaneously, the synapse attains its highest conductance, indicating optimal potentiation. In contrast, intermediate voltage amplitudes lead to intermediate conductance states. Lastly, when the voltage falls near the Neel-Brown threshold, around 150mV, typically due to a significantly delayed arrival of (*V*_*post*_) compared to (*V*_*pre*_), the synapse's likelihood to switch to a higher conductance state is almost negligible, demonstrating minimal potentiation. The same reasoning applies to synaptic depression. Based on the above explanation and the analysis of the orange and gray curves of Figures 5A, B, we set *the third design requirement as follows:*

- 1μ*s* < *W*_*post*_*max*_ < 10μ*s*: to modulate the switching probability from 0 to 100% in the applicable voltage amplitudes of depression.
- 1μ*s* < *W*_*post*_*min*_ < 10μ*s*: to modulate the switching probability from 0 to 100% in the applicable voltage amplitudes of potentiation.

We used the following values in this work: *W*_*post*_*max*_ = 1μ*s* and *W*_*post*_*min*_ = 7μ*s*.

**Figure 6:**
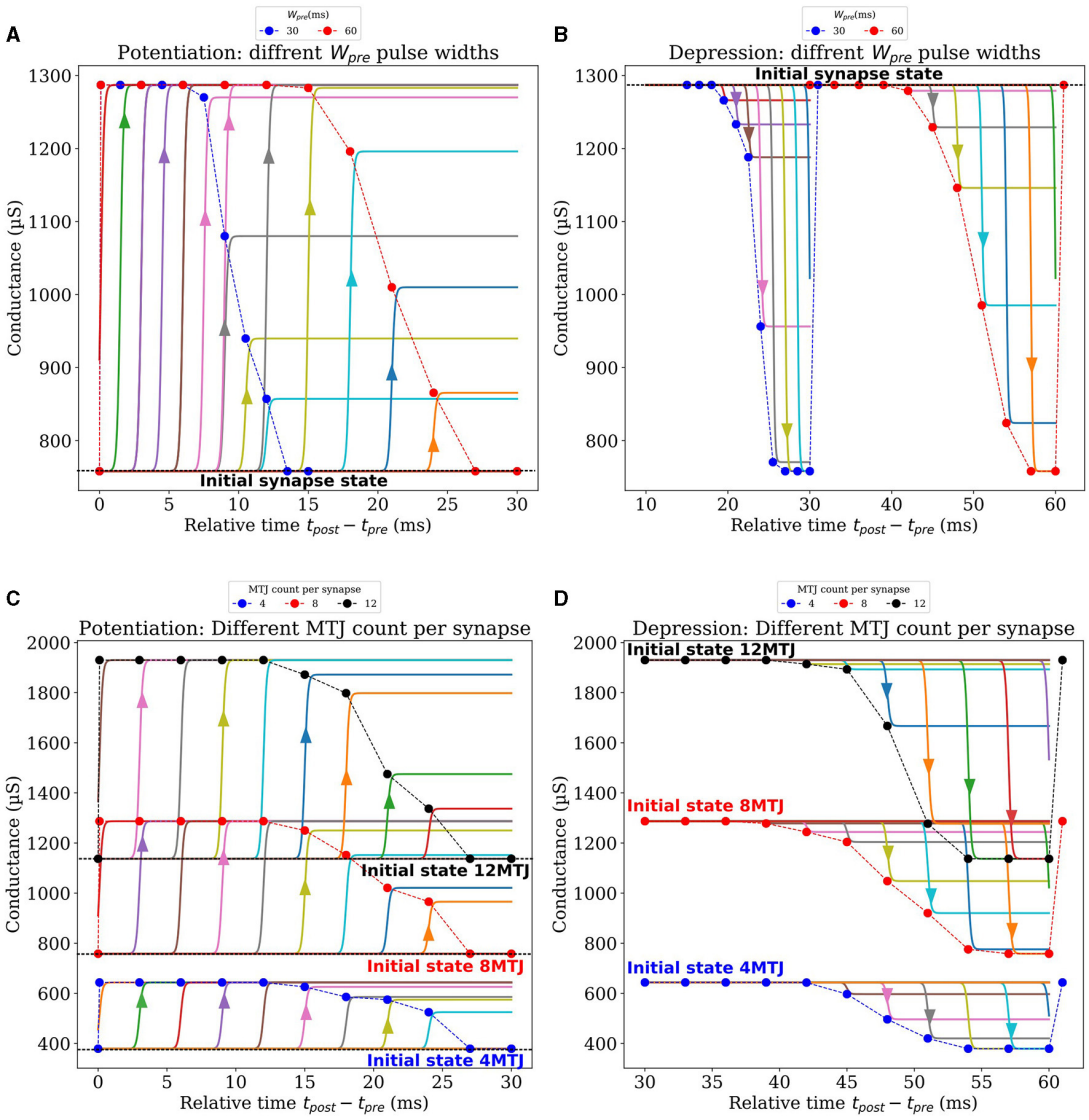
Synaptic design exploration based on electrical simulations. **(A, B)** Show the influence of two different pre-synaptic pulse widths on potentiation and depression curves respectively, for each *V*_*pre*_, *V*_*post*_ arrives at different delays, and the subsequent synaptic update is observed. **(C, D)** Present the effects of varying the number of MTJs per synapse in its conductance, for potentiation and depression respectively.

After discussing the post-synaptic pulse widths (*W*_*post*_*max*_ and *W*_*post*_*min*_ ), we focused on the pulse width of the pre-synaptic signal (*W*_*pre*_), which is on the order of milliseconds, to achieve frequencies within the kHz range, akin to those observed in biological STDP. Our approach aligns with the requirements of neuromorphic applications, which prioritize attributes such as energy efficiency over sheer speed. Examples of such applications include sensory data processing, decision-making under uncertainty, and learning from sparse data sets. The brain does note need high processing frequencies to perform these applications. Although the pulse width of *V*_*pre*_ does not directly influence MTJ switching probability due to being lower than the Neel-Brown threshold, it plays a crucial role in shaping the Bi-sigmoid learning curve, because the duration of the update interval corresponds to *W*_*pre*_. Through a series of Spice simulations, we explored various presynaptic pulses with different *W*_*pre*_ values, applied on a synapse composed of 8 MTJs. First, to explore the potentiation, all the MTJs of the synapse were initialized at a low conductance state. For each presynaptic pulse characterized by its width *W*_*pre*_, we run multiple independent simulations where at each simulation, *V*_*post*_ arrives at a given delay relative to *V*_*pre*_. Depending on the arrival time, the conductance of the synapse may be increased by a certain amount. This weight update is depicted in Figure 6A, where each point corresponds to a single arrival time of *V*_*post*_, and is obtained by averaging the conductance update of ten similar simulations to account for the MTJs' stochasticity. The synapse is then reset to the lowest conductance state, *V*_*post*_ arrives at a different delay, the update in conductance of ten similar simulations are averaged, and so on. After completing the sweep through *W*_*pre*_, the same analysis is carried out again for another presynaptic pulse with a different *W*_*pre*_. Figure 6A depicts the results for *W*_*pre*_ values of 30*ms* and 60*ms*, more curves for other presynaptic pulse widths can be found in the Supplementary material. It is important to highlight the re-initialization of the synapse to its lowest conductance state in our analysis after each occurrence of *V*_*post*_ at a certain delay. The arrows on Figure 6A highlight the instantaneous conductance increase at a given delay of *V*_*post*_ independently from other points. Consequently, the figure shows the clear effect of *V*_*post*_ when arriving at different delays relative to *V*_*pre*_. If they arrive simultaneously, the synapse reaches its highest conductance, whereas the increase of conductance (potentiation) gets lower as the delay increases, until *V*_*post*_ can no longer update the synapse at a substantial delay. Notably, the first point of each potentiation curve indicates the arrival of *V*_*post*_ before *V*_*pre*_. In such a case, no synaptic update should happen because the two signals are not overlapping, this explains why the synapse remains at its initialized state in this case. The same analysis was performed to study the effect of *W*_*pre*_ on the depression curves shown in Figure 6B. The synapse is initialized at the highest conductance state this time, the arrival of *V*_*post*_ at increasing delays increases the depression amplitude. As previously, after each occurrence of *V*_*post*_, the synapse is reset to the highest conductance state, *V*_*post*_ arrives at a different delay, the update in conductance of ten similar simulations are averaged, and so on. After completing the sweep through *W*_*pre*_, the same analysis is carried out again for another presynaptic pulse with a different *W*_*pre*_. More curves corresponding to different widths *W*_*pre*_ can be found in the Supplementary material. Similar to potentiation curves, the last point of each depression curve, where *V*_*post*_ arrives after *V*_*pre*_, show no synaptic change due to non-overlapping signals, maintaining the synapse at its initialized state. By making the potentiation and depression curves side by side, one can notice the region separating potentiation and depression where *V*_*post*_ has no effect on the synapse, for instance, this region extends from 27ms to 39ms in the case of *W*_*pre*_ = 60*ms*. Finally, The bottom line of this analysis, when comparing the effect of different widths (*W*_*pre*_), a smoother and more gradual transition occurs for larger widths, on both potentiation and depression. For the remaining of this paper, a width of 60*ms* is used for *W*_*pre*_.

#### 3.4.5 MTJ Count per Synapse

In our investigation of the impact of varying the number of MTJs per synapse, a similar analysis to the previous one was performed while fixing all pulse widths and amplitudes to the values justified earlier. Like the previous analysis, we vary the arrival time of *V*_*post*_ relative to *V*_*pre*_. After each occurrence of *V*_*post*_, the synapse is reset to its initial conductance state, *V*_*post*_ arrives at a different delay, the update in conductance of 10 similar simulations are averaged, and so on. After completing the sweep through *W*_*pre*_, the same analysis is carried out again with a synapse composed of a different number of MTJs. This process was repeated for synapse configurations containing 1 to 12 MTJs, and was carried out for synapses performing potentiation and depression. Figures 6C, D illustrate the result of this analysis for synapses composed of 4,8 and 12 MTJs. Extnesive analysis of synapses composed of other counts of MTJs can be found in Supplementary material. These figures display the synapse's updated conductance at each *V*_*post*_ arrival time, for both potentiation and depression phases, across the different MTJ counts per synapse. Notably, A higher count of MTJs per synapse was observed to enhance the resolution and the capacity of the synapse to hold a wider range of weight values. Such an increase in resolution and capacity is potentially advantageous for learning tasks that demand higher precision and nuanced weight adjustments. Finally, all the discussed parameters after optimization are summarized in Table 1.

**Table 1:** Summary of the design parameters.

	**Parameter**	**Description**	**Value**
Input spike	*V*_*pre*_*max*_	Voltage at the beginning	150 mV
	*V*_*pre*_*min*_	Voltage at the end	-90 mV
	*W*_*pre*_	Pulse width	60 ms
Output spike	*V*_*post*_*max*_	Voltage at the positive part	100 mV
	*W*_*post*_*max*_	Width at the positive part	1μ*s*
	*V*_*post*_*min*_	Voltage at the negative part	-100 mV
	*W*_*post*_*min*_	Width at the negative part	7μ*s*
Magnetic Tunnel Juncion MTJ		Thermal fluctuation dist	exponential
		Oxide thickness	8.5 Å
		Free layer thickness	1.3 nm
		total thickness of MTJ	33.55 nm
		MTJ radius	16 nm

## 4 Results and discussion

### 4.1 Derivation of Bi-Sigmoid STDP

The comprehensive design space exploration shown in the previous section led to the development of a novel learning rule for the synapse. This rule has an STDP-like behavior, where a short delay of *V*_*post*_ relative to *V*_*pre*_ leads to potentiation, and a longer delay results in depression. By analyzing the conductance changes in response to the relative time between pre- and post-synaptic spikes, we identified a pattern best represented by two sigmoid functions. This behavior was captured in a rule that we labeled *Bi-sigmoid STDP (B2STDP)* learning rule, formulated from electrical simulations data.

**Figure 7:**
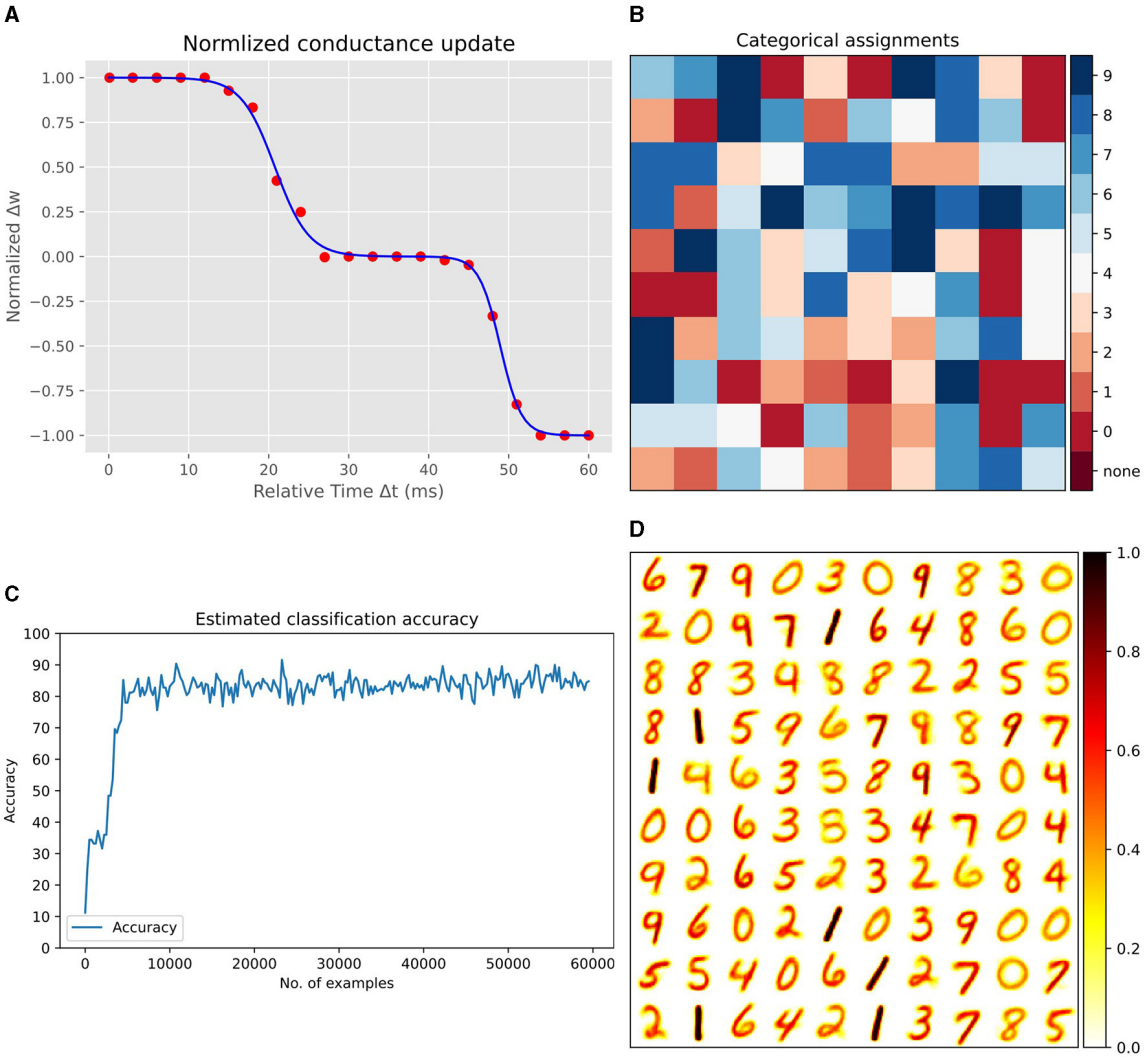
**(A)** Normalized synaptic weight updates relative to the timing differences between pre- and post-synaptic spikes. Spice simulation data fitted with the Bi-sigmoid function. **(B)** Label distribution map for each excitatory neuron after training, indicating learned representations. **(C)** Incremental improvement in classification performance as a function of the number of training samples. **(D)** Visualization of synaptic weights between the input layer and 100 excitatory neurons, reshaped into 28x28 matrices, each representing a learned digit from the MNIST dataset.

Figure 7A presents the results of our Spice simulations, which were conducted to determine the synaptic weight updates relative to the timing differences between pre- and post-synaptic spikes. In these simulations,we utilized a synapse model with 12 MTJs, with the parameters detailed in Table 1, and initialized the synapse to an intermediate conductance state. By varying the arrival time of *V*_*post*_ with respect to *V*_*pre*_, we record the resultant changes in synaptic conductance. After each sweeping step of *V*_*post*_, the synapse was reset to its initial state. This process was repeated 10 times, and the conductance updates were then averaged and normalized. The fitting of this data to a Bi-sigmoid function, as depicted in the figure, accurately represents the updates in synaptic conductance. The fitting function is given by:


$$\begin{array}{l} \Delta w\;\left(\Delta t\right)=\frac{-\frac{A}{1+e^{-k_{0}\left(\Delta t-t_{0}\right)}}-\frac{A}{1+e^{-k_{1}\left(\Delta t-t_{1}\right)}}+A}{A} \end{array}$$

where Δ*w* is the normalized synaptic update and Δ*t* is the delay between pre- and post-synaptic spikings, and the following are fitting constants:


$$\begin{array}{r} A=7.95\times 10^{5};\;k_{0}=0.474723045;\\ t_{0}=20.77893753;\;k_{1}=0.757072031;\;t_{1}=48.93860322 \end{array}$$

The B2STDP is distinguished from classical STDP by being intrinsically tied to the physical properties of MTJs, enabling on-the-fly synaptic updates based on spiking activity, thus promoting local learning with in-memory computing without the need to store the spiking history of neurons (traces) in an external memory. More details on the derivation of the Bi-sigmoid rule can be found in the Supplementary material.

### 4.2 Integration in a functional network

To evaluate our B2STDP learning rule in an image classification task, we integrated it into Bindsnet Hazan et al. (2018); an SNN simulation framework (Supplementary material provides detailed steps on integrating our learning rule in the SNN framework). We used the SNN architecture introduced in Diehl and Cook (2015). This network utilizes a two-layer structure: the first layer consists of 784 input neurons, corresponding to the 28 × 28 pixels of the MNIST images (LeCun et al., 1989), and the second layer comprises 100 excitatory and an equal number of inhibitory neurons. The network employs leaky integrate-and-fire neuron models and conductance-based synapses. Inputs are presented as Poisson spike trains, with firing rates proportional to pixel intensities, converting the intensity values of MNIST images into spikes. The synapses between input and excitatory neurons learn according to B2STDP, which allows not only unsupervised learning but also neuromorphic efficiency through in-memory computing thanks to MTJ-based synapses.

### 4.3 Network performance

During training, each input image is converted into spikes proportional to the pixel intensities and shown to the network for 250*ms*. For each output neuron, the network keeps track of how many spikes it produces in response to each class of input. After exposing the network to the complete training dataset, we calculate the average firing rate for each neuron for every class. The class that causes the highest average firing rate for a particular neuron becomes that neuron's assigned label. Figure 7B displays the assigned label for each excitatory neuron after training. This label distribution map showcases the learned representations across the excitatory layer. Notably, the assignment of labels is based on the predominance of neuron firing in response to specific input classes throughout the training phase. During evaluation, the accuracy is calculated by counting all the spikes from excitatory neurons which were all assigned a label during training, and seeing which class gets the most spikes for each input. Each neuron 'votes' for its assigned class every time it fires. The class with the most votes across all neurons is the predicted class for that input. The network's accuracy is measured by how often the predicted class matches the actual class of the inputs. Figure 7C, demonstrates the incremental improvement in classification performance as the network processes a greater number of training samples.

Finally, Figure 7D presents a visualization of the synaptic weights between the input layer and the excitatory neurons in the network. Each weight vector, originally 784-dimensional corresponding to the flattened 28 × 28 pixel MNIST images, is reshaped back into a 28 × 28 matrix. The figure illustrates 100 such matrices, each corresponding to a different excitatory neuron in the network. These matrices serve as a snapshot of what each neuron has learned to recognize, with each matrix visually resembling a digit from the dataset. This underscores the network's ability to extract key features from the training data.

The results presented in Table 2 demonstrate the effectiveness of our B2STDP learning rule within SNNs, showcasing a competitive performance compared to other notable works. In our experiments, we trained networks with different numbers of output neurons - specifically 100, 400, and 1,600–over 1, 3, and 3 epochs, respectively. The networks achieved testing accuracies of 85.15%, 90.28%, and 91.71% for each neuron count. Remarkably, with 100 output neurons, our network surpasses the classification accuracies of both Diehl and Cook (2015) and Tang and Gao (2023) implementations. Although increasing the number of output neurons does improve accuracy, the enhancement is not dramatic, likely due to the need for additional epochs to achieve convergence in accuracy. Nonetheless, our model still outperforms (Tang and Gao, 2023) and attains comparable results to Diehl and Cook (2015), while requiring fewer epochs. For instance, where Tang and Gao (2023) and Diehl and Cook (2015) employed 4 and 7 epochs to train the SNN with 1,600 neurons, our network needed only 3 epochs to reach similar levels of accuracy. Two adjustments may have contributed to the performance of our network: the duration for which an image is presented to the network, and the conversion coefficient for translating pixel intensity into firing rates. By fine-tuning these parameters to 250ms for image presentation and setting a maximum firing rate at 60 Hz, our network demonstrates enhanced efficiency and accuracy. While traditional ANNs employing backpropagation may achieve higher accuracies, our approach, centered on an unsupervised learning paradigm, offers significant advantages for neuromorphic hardware implementations, particularly in terms of energy efficiency, online and unsupervised learning which are important for IoT devices. Central to our approach is the adoption of a novel learning rule that is intrinsically tied to the physical properties of spintronic synapses.

**Table 2:** Comparison of classification accuracy across different works and network sizes.

**Neurons**	**100**	**400**	**1,600**
Diehl and Cook (2015)	82.9 %	87.0 %	91.9 %
Tang and Gao (2023)	75 %	83 %	88 %
**This work**	85.15 %	90.28 %	91.71 %

Our study primarily focused on optimizing synapse behavior in accordance with MTJ dynamics, revealing several areas for potential future exploration. Enhancing accuracy is an ongoing objective, with improvements potentially arising from adjustments in network parameters such as neuron counts, spiking thresholds, and the balance between excitatory and inhibitory neurons. It is crucial to note that our approach with SNNs is not about outperforming DNNs in accuracy; rather, it emphasizes the unique advantages of SNNs, especially empowered by unsupervised and online learning, while being known for their energy efficiency, making them well-suited for IoT devices. Issues like dealing with leakage currents in the crossbar, significant in design and optimization phases, should be further investigated to optimize power efficiency. Reliability concerns related to MTJ variability and defects, such as pinhole defects and dielectric breakdown, are active research topics when MTJs are used as MRAMs. Their use as synapses warrants further examination to understand how these issues affect the learning rule and, subsequently, the overall performance of the SNN. Additionally, developing a comprehensive network that includes digital neuron designs integrated with crossbar arrays may facilitate an accurate evaluation of power consumption in SNNs employing the bisigmoid learning rule. The choice of dataset is another important aspect, where data originally obtained in a spiking manner, like images from event-based DVS cameras, are better suited for SNNs than images converted to spikes. These potential refinements represent promising avenues to elevate the capabilities of our neuromorphic computing model, potentially achieving higher accuracies and more efficient learning processes.

## 5 Conclusion

In this study, we explored the potential of MTJs to create efficient spintronic synapses for SNNs, utilizing multiple MTJs in parallel to form a proposed synapse. By operating the synapse at low voltages and exploiting the stochastic nature of MTJs, we enable the synapse to achieve multiple conductance levels. These levels are attained through training, guided by the novel Bi-sigmoid STDP learning rule. This rule, facilitated by engineered linearly decaying presynaptic and bi-rectangular postsynaptic pulses, translates the delay between the two pulses into voltage modulation, effectively updating the synapse state. The resultant rule operating in the proposed synapse will enable energy-efficient neuromorphic systems capable of supporting unsupervised and online learning, eliminating the need for labor-intensive data labeling and enabling continuous learning after deployment. Through detailed electrical SPICE simulations, we optimized the MTJ-based synapse design, demonstrating how different pulse widths and synapse configurations influence the Bi-sigmoid rule. Finally, the integration of this rule in an SNN led to a notable 91.71% accuracy in unsupervised image classification. While this work has centered on synaptic mechanisms, addressing future challenges such as leakage currents, reliability concerns including MTJ variability and defects, and further tuning of neuron and network parameters, alongside exploring datasets suited for spiking data, presents promising pathways for enhancing system performance and advancing neuromorphic computing development.

## Data availability statement

Simulation data can be made available upon reasonable request from the authors. Bi-sigmoid rule integration in Bindsnet can be found here: https://github.com/salah-daddi-nounou/bindsnet/blob/my_changes/examples/mnist/salah_example.pys.

## Author contributions

SD: Conceptualization, Data curation, Investigation, Methodology, Software, Writing – original draft, Writing – review & editing. E-IV: Conceptualization, Funding acquisition, Project administration, Supervision, Writing – review & editing.
