# Supplementary material for: Bi-sigmoid spike-timing dependent plasticity learning rule for magnetic tunnel junction-based SNN
Source: Front Neurosci. 2024 May 15;18:1387339. doi: 10.3389/fnins.2024.1387339 (PMC11137280; doi:10.3389/fnins.2024.1387339)
Supplement: Supplementary file 1 [file Data_Sheet_1.PDF]

Bi-Sigmoid Spike-Timing Dependent Plasticity Learning Rule for Magnetic Tunnel Junction-based SNN

Supplementary Material

Salah DADDINOUNOU*[1] and Elena-Ioana VATAJELU[1]

[1]Univ. Grenoble Alpes, Grenoble INP, TIMA, 38000 Grenoble, France
Email: {salah.daddinounou, ioana.vatajelu}@univ-grenoble-alpes.fr

1 Extinsive SPICE simulations

For potentiation, the synapse is re-initialized to its lowest conductance each time. The arrival of V_{post} at short delays compared to V_{pre} causes the synapse to potentiate (increase its conductance) by different amounts. Importantly, the first point of any potentiation curve shows the case where V_{post} arrives before V_{pre} where the two signals don't overlap. In this case the synapse stays at its initialized state (lowest conductance). For depression, the synapse is re-initialized to its highest conductance each time. The arrival of V_{post} at long delays compared to V_{pre} causes the synapse to depress (decrease its conductance) by different amounts. Importantly, the last point of any depression curve shows the case where V_{post} arrives after V_{pre} where the two signals don't overlap. In this case the synapse stays at its initialized state (highest conductance).

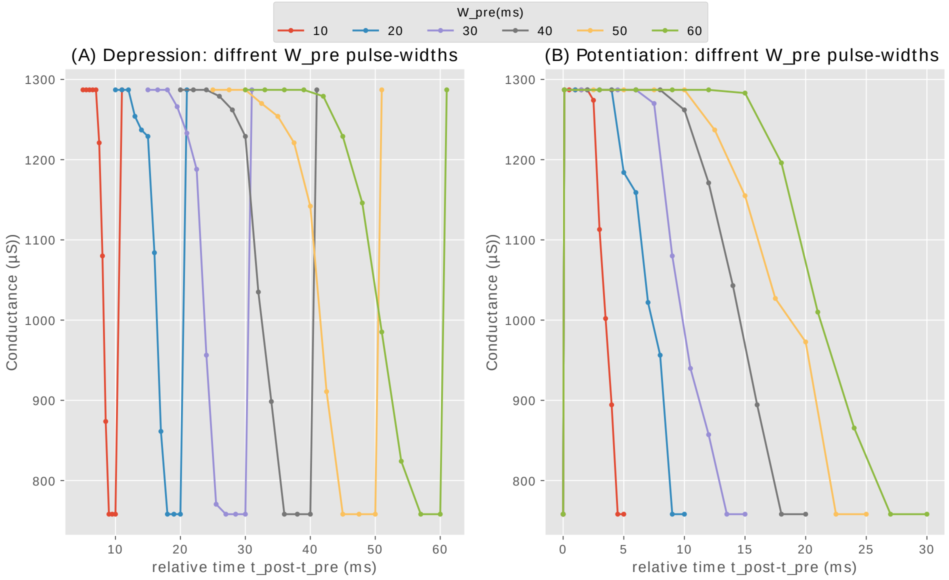

Figure 1: Electrical simulations show the influence of different pre-synaptic pulse widths on potentiation and depression curves respectively, for each V_{pre}, V_{post} arrives at different delays, and the subsequent synaptic update is observed.**(A)**:Depression, **(B)**:Potentiation.

Figure 1 and Figure 2 display the effect of different presynaptic pulse widths, and synapse configurations respectively on the weight update. Figure 3 breaks down one of the curves of

*Corresponding author: salah.daddinounou@univ-grenoble-alpes.fr

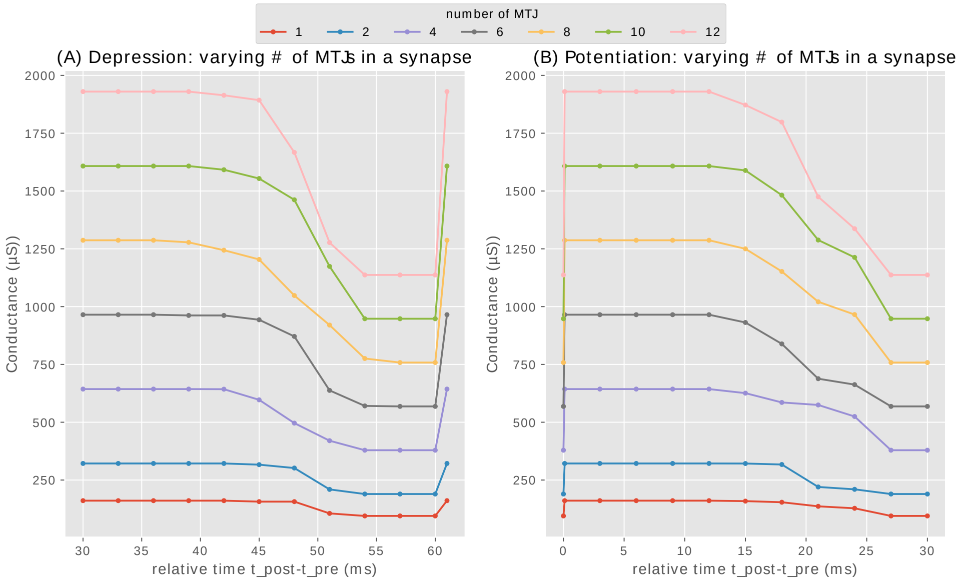

Figure 2: Electrical simulations show the effects of varying the number of MTJs per synapse in its conductance. **(A)**:Depression, **(B)**:Potentiation.

Figure 2 (12MTJ; $W_{pre} = 60ms$) and shows the 10 simulation iterations for each delay (to account of MTJs stochasticity) and displays the corresponding average curve. Each curve of Figure 1 and Figure 2 is the result of the same averaging process.

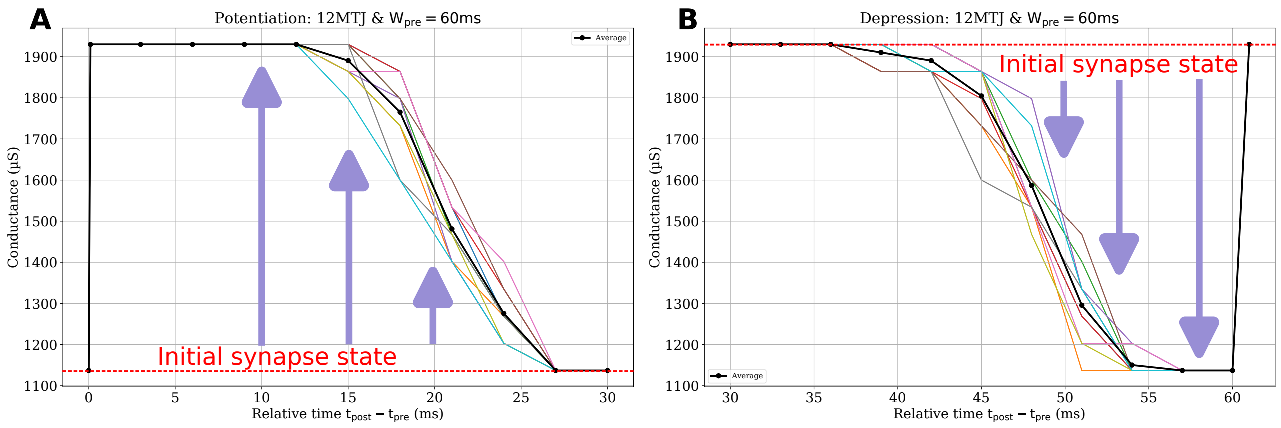

Figure 3: The synaptic update of conductance as a function of the delay between pre- and post pulses. Each point is the result of averaging ten simulations. **(A)**: Potentiation, **(B)**: Depression

2 Bi-sigmoid rule Derivation

The derivation of the Bi-sigmoid learning rule from simulation data is succinctly outlined in the following steps:

1. **SPICE Simulation:** We utilized the outcomes from SPICE simulations of a 12MTJ-based synapse under potentiation and depression conditions, as depicted in Figure 3. Each data point represents the average of 10 similar simulations, accounting for the intrinsic stochasticity of MTJ devices.
2. **Data Refinement:** To accurately reflect the STDP mechanism, we excluded the first point of potentiation and the last point of depression, where V_{post} either precedes V_{pre} in potentiation or follows V_{pre} in depression, as these instances do not contribute to overlapping

signal-induced synaptic changes. The exact conductance update was then determined by calculating the difference between the final and initial conductance states.

3. **Integration and Fitting:** We combined potentiation and depression data into a single plot and applied a bi-sigmoid fitting:

$$\Delta w(\Delta t) = \frac{-\frac{A}{1+e^{-k_0(\Delta t - t_0)}} - \frac{A}{1+e^{-k_1(\Delta t - t_1)}} + A}{A}$$

   Parameters: $A = 7.95 \times 10^5$, $k_0 = 0.474723045$, $t_0 = 20.77893753$, $k_1 = 0.757072031$, $t_1 = 48.93860322$.
   Please note that the fitting parameters were obtained with values of conductance in nanoSiemens (nS) and time in milliseconds (ms).

4. **Normalization:** Finally, the conductance updates and the fitting function were normalized to standardize the bi-sigmoid STDP with other synapse configurations. Figure 4 displays the combined simulation points of potentiation and depression (normalized conductance values), The points are fitted with the above function.

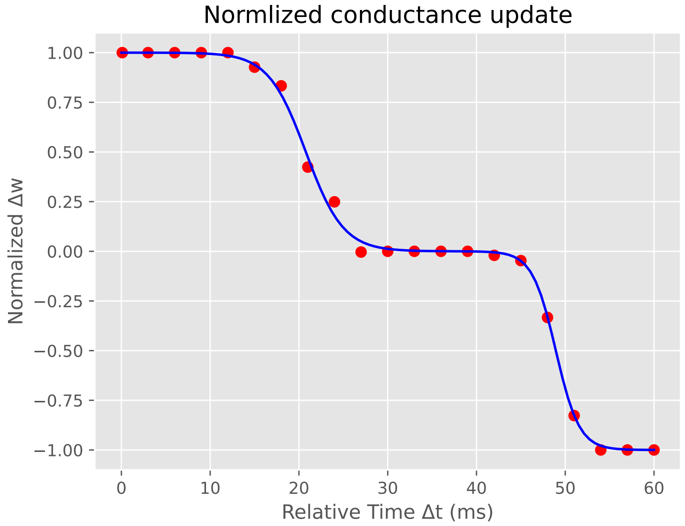

Figure 4: Normalized simulations data of potentiation and depression of 12MTJ-based synapse fitted by a bi-sigmoid function.

3 Bi-sigmoid rule integration in Bindsnet

Our work involved extending the BindsNET framework to incorporate the Bi-sigmoid STDP rule. The source code modifications and the new implementation are available in our fork of the BindsNET repository, which can be found in our GitHub Repository [1]. The process of integrating the Bi-sigmoid STDP rule into the BindsNET framework is as follows:

1. We developed a custom training script named `salah_example.py`, inspired by the Bindsnet example `eth_mnist.py`. This script trains the SNN and subsequently saves the trained weights and network parameters as PyTorch objects (‘.pt‘ files).

2. Within the `nodes.py` base class, we introduced a new trace, `x2`, defined by the `bisigmoid_trace(t_)` function. We added the trace `x2` to the block that manages the traces, enabling all neuron types to record this novel trace as they spike.

[1] https://github.com/salah-daddi-nounou/bindsnet/tree/my_changes

3. In `learning.py`, the Bi-sigmoid learning rule is introduced, updating synaptic weights based on the `x2` trace. Here how this rule is used to update weights in the network:
   - **Trace Recording**: Both input and output neurons record two key traces: `x2`, defined by the bisigmoid function, and `s`, indicating spikes. Here, `source` represents the input neuron while `target` refers to the output (excitatory) neuron.
   - **Rule Application**: The Bi-sigmoid learning rule is applied exclusively when the output neuron (`target.s`) spikes. At this moment, the connection between the input and output neurons is updated based on the value of `source.x2` at the time of `target.s` spiking.
4. A new network model, `Salah_model`, was introduced in `models.py`, similar to the model `DiehlAndCook2015` but notably incorporating the Bi-sigmoid rule as the `update_rule`.
5. Finally, For the purpose of inference, `evaluate_plot.py` was created. This script facilitates the loading of trained weights, performs model evaluation, and generates plots to visually represent the network's performance post-training.

Table 1: MTJ Parameters

Parameter	Description	Value
α	Gilbert Damping Coefficient	0.027
P	Electron Polarization Percentage %	52
H_k	Out of plane Magnetic Anisotropy (Oe)	1433
M_s	Saturation Field in the Free Layer (Oe)	15800
r	Radius of the MTJ nanopillar (nm)	16
t_{sl}	Height of the Free Layer (nm)	1.3
t_{ox}	Height of the Oxide Barrier (nm)	0.85
$thick_s$	Total Thickness of th MTJ (nm)	33.55
TMR	TMR(0) with Zero Volt Bias Voltage %	70
T	Temperature (K)	300
RA	Resistance area product ($\Omega\mu m^2$)	5
	Thermal fluctuation distribution	exponential
